# Non-Surgical Cancer Risk Reduction in *BRCA1* Mutation Carriers: Disabling the Remote Control

**DOI:** 10.3390/cancers12030547

**Published:** 2020-02-27

**Authors:** Martin Widschwendter, Louis Dubeau

**Affiliations:** 1 Department of Women’s Cancer, University College London, 74 Huntley Street, London WC1E 6AU, UK; 2 Research Institute for Biomedical Aging Research, Universität Innsbruck, 6020 Innsbruck, Austria; 3 European Translational Oncology Prevention and Screening (EUTOPS) Institute, 6060 Hall in Tirol, Austria; 4 Department of Pathology, Keck School of Medicine, USC/Norris Comprehensive Cancer Centre, University of Southern California, Los Angeles, CA 90089, USA

**Keywords:** BRCA1 mutations, ovarian cancer, breast cancer, prevention

## Abstract

Women-specific cancers are a major health issue, particularly those associated with the *BRCA1* germline mutation carrier state, which include triple-negative basal breast carcinomas and high-grade serous ovarian carcinomas (referred to as extra-uterine Müllerian carcinomas). Whereas many chronic diseases can currently be prevented (e.g., cardiovascular diseases), no recent tangible progress was made in cancer prevention of *BRCA1* mutation carriers apart from surgical resections of at-risk organs. This lack of progress is largely due to (1) poor understanding of the initiating events triggered by known risk factors in the development of these cancers, (2) the fact that current preventive measures rely on evidence obtained from adjuvant breast cancer treatment that fail to protect against poor prognostic cancers, and (3) problems with using cancer incidence in high-risk women as an ethically justifiable endpoint in cancer prevention trials. Here, we propose that cancer predisposition in *BRCA1* mutation carriers is driven, at least in part, by cell-nonautonomous mechanisms (i.e., driven by consequences of this carrier state on hormonal and other systemic factors controlled in organs other than those that are cancer-prone) and that biomarkers of epigenomic reprogramming, hypothesized to be a direct consequence of such cell-nonautonomous mechanisms, are attractive as intermediate surrogate endpoints to assess the efficacy of cancer risk-reducing strategies targeting these mechanisms.

## 1. Introduction

Surgical resection of ovaries, fallopian tubes, and breasts is currently the most effective means of cancer risk reduction in individuals with germline *BRCA1* mutations. Emotional, physiological, and reproductive consequences of these procedures lead a significant proportion of mutation carriers to either delay or refuse these potentially life-saving interventions, hence a need for effective non-surgical means of risk reduction. There are currently no prospectively validated non-surgical measures to prevent cancers of the reproductive tract in *BRCA1* mutation carriers. Current non-surgical measures of breast cancer prevention using tamoxifen or aromatase inhibitors rely on evidence gained from adjuvant treatment and offer poor protection against the triple-negative subtype typically associated with the *BRCA1* mutation carrier state [1,2].

**Figure 1:**
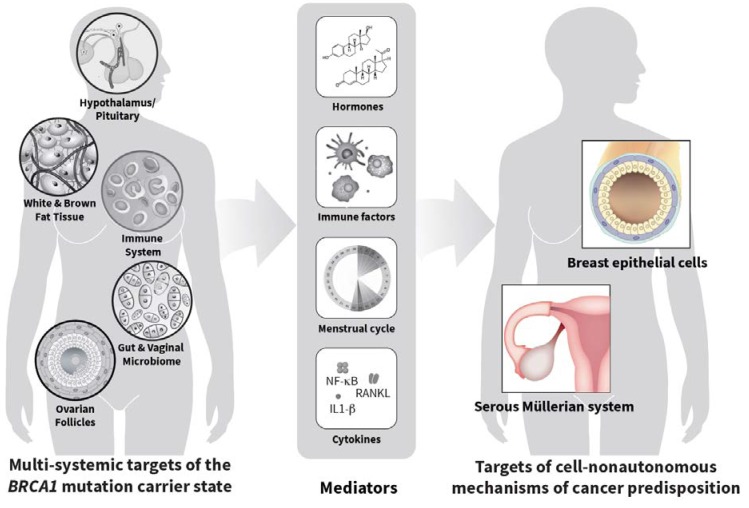
Cell-nonautonomous mechanisms of breast and ovarian cancer predisposition in *BRCA1* mutation carriers. The diagram on the left illustrates various organs or systems other than those carrying an elevated cancer risk that are influenced by the *BRCA1* mutation carrier state. These multi-systemic effects lead to alterations in levels of hormones and cytokines such as those referred to as “mediators” in the figure. Such alterations interplay with cell-autonomous effects of the *BRCA1* mutation carrier state resulting in increased cancer risk in the organs shown on the right.

Effective non-surgical approaches for cancer risk-reduction in *BRCA1* mutation carriers will likely come from thorough understanding of the mechanisms responsible for cancer predisposition in this population. BRCA1 is widely regarded as a classical tumor suppressor, implying that loss of its function in a given cell directly leads to increased risk of malignant transformation in that particular cell. Progress in understanding the consequences of *BRCA1* mutations on DNA repair pathways evolved from this concept and had a significant impact on the treatment of cancers associated with the *BRCA1* mutation carrier state [3]. It was suggested that targeting poly (ADP ribose) polymerase (PARP) might have merit as a risk reduction measure [4]. Here, we seek to bring attention to the existence of multi-systemic consequences of the *BRCA1* mutation carrier state that do not directly target tissues that are at elevated cancer risk in carriers of such mutations, but alter homeostasis in tissues that influence Müllerian and mammary epithelia from a distance (cell-nonautonomously) through hormones or cytokines released in the blood circulation (Figure 1). 

Strong support for the idea that events originating outside the tissues that are at elevated cancer risk in *BRCA1* mutation carriers are important drivers of such risk comes from the well-established association between menstrual cycle activity and incidence of serous carcinomas of the Müllerian tract. Indeed, interruption of the menstrual cycle by either pregnancy or oral contraceptive use has a profound effect on the life-time risk of these cancers not only in the general population, but also in *BRCA1* mutation carriers [5,6,7,8]. Additional evidence comes from work with genetically modified experimental animals. Introducing a conditional *Brca1* mutation specifically targeting mouse ovarian granulosa cells, the main site of sex steroid hormone biosynthesis during reproductive years, leads to benign tumors resembling serous cystadenomas in the para-ovarian and para-tubal areas [9]. The incidence of these tumors correlates with the magnitude of consequences of such mutations on estrous cycle homeostasis, which include prolongation of the estrogen-dominant pre-ovulatory phase and increased circulating levels of estradiol [10]. The physiological significance of these observations is underscored by evidence of increased estrogen stimulation in organs targeted by this hormone in mice harboring Brca1-deficient ovaries including increased proliferative index in the endometrium during the pre-ovulatory phase of the estrous cycle and increased length of long bones [10,11]. Such multi-systemic effects, although more severe in mice that carry a homozygous *Brca1* mutation in their ovarian granulosa cells, are also significant in animals that carry a heterozygous mutation such as present in human *BRCA1* mutation carriers [11,12]. The relevance of these observations in experimental animals to human *BRCA1* mutation carriers is supported by findings of increased endometrial thickness during the proliferative phase of the menstrual cycle in such carriers, as well as of higher levels of circulating sex steroid hormones [12]. These observations are intriguing given the well-established consequences of menstrual cycle activity on extra-uterine Müllerian and breast carcinomas in the general population [5,6,7,8]. 

Alterations in menstrual cycle homeostasis in *BRCA1* mutation carriers may account for the increased activity of the *RANKL/RANK/OPG* signaling pathway previously reported in such mutation carriers [13]. RANKL (Receptor activator of nuclear factor kappa-Β ligand), the molecular trigger of this pathway, is induced by progesterone [14], a menstrual hormone that is elevated in the blood of *BRCA1* mutation carriers compared to non-carriers [12]. Activation of this pathway not only results in increased proliferation of basal cells in mammary ductal epithelium, but also leads to mammary cancer predisposition in a rodent model [13,14,15], underscoring a potentially important cell-nonautonomous scenario contributing to breast cancer predisposition in *BRCA1* mutation carriers. Specific molecular events similarly driving cell-nonautonomous mechanisms of cancer predisposition to Müllerian cancers of the reproductive tract remain to be identified and are likely different than those underlying breast cancer predisposition because of differences in the nature of the sex steroid hormones that drive these cancers. Indeed, Müllerian carcinomas are estrogen-driven while mammary carcinomas are progesterone-driven. This may account for the fact that, although parity and oral contraceptive use are strongly protective against extra-uterine Müllerian carcinomas even in *BRCA1* mutation carriers [5,16,17,18,19], oral contraceptives appear to increase risk of at least certain subtypes of breast cancer in at least certain segments of the general population, while their effect on breast cancer risk in *BRCA1* mutation carriers is unclear [7,8,20,21]. Nevertheless, we propose that, similarly to the mammary gland, hormonal characteristics associated with menstrual cycle progression in *BRCA1* mutation carriers lead to increased proliferative activity in specific components of the Müllerian tract of such carriers including but not limited to the fallopian tube fimbriae [22].

Cancers associated with the *BRCA1* mutation carrier state invariably harbor *TP53* mutations that are present not only in mature tumors, but also in their precursor lesions [23]. Reduction of intracellular BRCA1 to levels similar to those present in *BRCA1* mutation carriers in an *in vitro* cell culture model, when accompanied by loss of TP53 function, leads to mitotic errors resulting in polyploidy/aneuploidy [4,24]. These changes are only significant in cells that reach a high replicative age (i.e., age based on number of prior cell divisions instead of chronological age) [4,24]. Thus, consequences of the *BRCA1* mutation carrier state on menstrual cycle homeostasis leads, via cell-nonautonomous mechanisms, to accelerated replicative aging (a consequence of increased proliferative activity) in specific segments of the reproductive tract. We hypothesize that the resulting high replicative age triggers cell-autonomous effects of this carrier state on mitotic homeostasis, resulting in aneuploidy and malignant transformation. Work with experimental animals suggests that accelerated replicative aging in the fallopian tubes of *BRCA1* mutation carriers may be driven not only by increased proliferation rate, but also by prolongation of the pre-ovulatory phase of the menstrual cycle, which is clearly associated with increased proliferation in this epithelium [10]. 

The elements of our hypothesis are shown in Figure 1, which also illustrates the possibility that systemic consequences of the *BRCA1* mutation carrier state may not be limited to menstrual cycle homeostasis. Other physiological processes or systems such as the immune system [25], fat metabolism [26], etc., all of which could potentially contribute to cancer risk, may also be influenced by this carrier state. Indeed, variations in immune function during the menstrual cycle, especially in sexually active women, are well documented [27,28,29] while a DNA methylation signature in blood cells, but not in buccal cells, is associated with cancer risk [25]. Moreover, lipid and metabolite deregulation was reported in breasts of *BRCA1/2* mutation carriers [26]. The proportion of women with a non-lactobacillus-dominated vaginal microbiome is significantly higher in women with extra-uterine Müllerian carcinomas and in yet unaffected *BRCA1* mutation carriers than in unaffected non-carriers [30]. This attests to the potential complexity and multi-systemic involvement of cancer predisposition in these carriers. Effects of a conditional *Brca1* mutation confined to ovarian granulosa cells on olfaction, an important driver of reproductive functions, further underscore this complexity [31].

Targeting cell-nonautonomous effects of the *BRCA1* mutation carrier state such as those illustrated in Figure 1 could become the basis of non-surgical means of risk reduction. This idea is attractive because, by definition, such mechanisms are mediated by factors present in the blood circulation and are, thus, more easily targetable than intra-cellular pathways. A better understanding of the circulating factors mediating cell-nonautonomous effects of the *BRCA1* mutation carrier state should also lead to the identification of novel biomarkers of cancer risk predisposition, facilitating evaluation of cancer risk in germline mutation carriers. This would be especially useful in the management of individuals with germline mutations of unknown significance. Such insights should also lead to the identification of novel biomarkers that may be useful as intermediate surrogates of responsiveness to treatment and intermediate endpoints in cancer prevention trials. Using cancer incidence as an endpoint for cancer prevention trials targeting *BRCA1* mutation carriers may raise ethical concerns due to unrealistically long study periods or prohibitive costs. The availability of intermediate endpoint biomarkers would alleviate this concern. Using biomarkers associated with systemic consequences of the *BRCA1* mutation carrier state would also raise the possibility of measuring such biomarkers not only in risk-prone tissues, but also perhaps in more readily accessible tissues such as, for example, cervical epithelium, buccal cells, or blood, further facilitating the design and execution of cancer prevention trials [32]. A thorough understanding of the multi-systemic consequences of the *BRCA1* mutation carrier state, including how these consequences are altered as a consequence of age or menopausal status, and of their cell-nonautonomous effects on the tissues at elevated cancer risk in such carriers would accelerate progress toward these goals.

Epigenetic reprogramming of the fallopian tube fimbrial epithelium may be a direct consequence of cell-nonautonomous effects of the *BRCA1* mutation carrier state on fimbrial epithelium [33]. The merit of using this epigenetic signature as surrogate intermediate endpoints is supported by a study of more than 1000 healthy female volunteers where aspirin, a drug known to reduce ovarian cancer risk [34], led to a substantial reduction of DNA methylation in genetic loci encoding mediators of the cancer-associated stem-cell phenotype [35]. This effect of aspirin was also detectable in readily accessible normal surrogate tissues [36]. A different panel of epigenetic biomarkers called epiTOC, which is specifically associated with replicative (as opposed to chronological) age, was also characterized [37]. Given that accelerated mitotic aging is hypothesized to be a direct consequence of the cell-nonautonomous effects of the *BRCA1* mutation carrier state on extra-uterine Müllerian epithelium, this panel might also represent an attractive biomarker of response to non-surgical means of cancer prevention.

## 2. Conclusions

We propose that targeting systemic effects of the *BRCA1* mutation carrier state that indirectly contribute to breast and Müllerian cancer risk elevation in such carriers is a potentially attractive approach toward the important goal of developing effective non-surgical means of cancer risk reduction in this population. Mediators of such cell-nonautonomous mechanism of cancer predisposition could also represent useful biomarkers to facilitate overall evaluation and clinical management of *BRCA1* mutation carriers. These biomarkers could even facilitate evaluation of cancer risk in the general population because menstrual cycle homeostasis, which is an important driver of cell-nonautonomous mechanisms of cancer predisposition in *BRCA1* mutation carriers, is the greatest known risk factor for the main histological subtypes of sporadic (non-familial) Müllerian carcinomas.
